# *Klebsiella pneumoniae* Susceptibility to Carbapenem/Relebactam Combinations: Influence of Inoculum Density and Carbapenem-to-Inhibitor Concentration Ratio

**DOI:** 10.3390/biomedicines10061454

**Published:** 2022-06-20

**Authors:** Maria V. Golikova, Kamilla N. Alieva, Alla V. Filimonova, Vladimir A. Ageevets, Ofeliia S. Sulian, Alisa A. Avdeeva, Sergey V. Sidorenko, Stephen H. Zinner

**Affiliations:** 1 Department of Pharmacokinetics & Pharmacodynamics, Gause Institute of New Antibiotics, 11 Bolshaya Pirogovskaya Street, 119021 Moscow, Russia; qvimqwem@yandex.ru (K.N.A.); allafil@yandex.ru (A.V.F.); 2 Pediatric Research and Clinical Center for Infectious Diseases, 9 Prof. Popov Street, 197022 Saint Petersburg, Russia; ageevets@list.ru (V.A.A.); sulyan1994@mail.ru (O.S.S.); avdeenko-alya@mail.ru (A.A.A.); sidorserg@gmail.com (S.V.S.); 3 Department of Medical Microbiology, North-Western State Medical University Named after I.I. Mechnikov, 195067 Saint Petersburg, Russia; 4 Harvard Medical School, Department of Medicine, Mount Auburn Hospital, 330 Mount Auburn St., Cambridge, MA 02138, USA; szinner@mah.harvard.edu

**Keywords:** inoculum effect, beta-lactams, beta-lactamase inhibitors, imipenem, doripenem, relebactam, *Klebsiella pneumoniae*

## Abstract

The inoculum effect (IE) is a well-known phenomenon with beta-lactams. At the same time, the IE has not been extensively studied with carbapenem/carbapenemase inhibitor combinations. The antibiotic-to-inhibitor concentration ratio used in susceptibility testing can influence the in vitro activity of the combination. To explore the role of these factors, imipenem/relebactam and doripenem/relebactam MICs were estimated against six *Klebsiella pneumoniae* carbapenemase (KPC)-producing *Klebsiella pneumoniae* strains at standard inocula (SI) and high inocula (HI) by two methods: with a fixed relebactam concentration and with a fixed, pharmacokinetic-based carbapenem-to-relebactam concentration ratio. The combination MICs at HI, compared to SI, increased with most of the tested strains. However, the IE occurred with only two *K. pneumoniae* strains regardless of the MIC testing method. The relationship between the MICs at SI and the respective inoculum-induced MIC changes was observed when the MICs were estimated at pharmacokinetic-based carbapenem-to-relebactam concentration ratios. Thus, (1) IE was observed with both carbapenem/relebactam combinations regardless of the MIC testing method; however, IE was not observed frequently among tested *K. pneumoniae* strains. (2) At HI, carbapenem/relebactam combination MICs increased to levels associated with carbapenem resistance. (3) Combination MICs determined at pharmacokinetic-based carbapenem-to-inhibitor concentration ratios predict susceptibility elevations at HI in KPC-producing *K. pneumoniae*.

## 1. Introduction

Beta-lactam/beta-lactamase inhibitor combinations are widely used to treat infections caused by Gram-negative bacteria that produce beta-lactamases. Imipenem/relebactam is a recently approved combination effective against carbapenem-resistant *Klebsiella pneumoniae* carbapenemase (KPC)-producing *Klebsiella pneumoniae* [1,2,3]. As was shown in numerous in vitro studies, relebactam effectively restores the antibacterial activity of imipenem against carbapenem-resistant bacteria [4,5,6]. However, it is not yet clear whether the antibacterial activity of imipenem/relebactam is maintained at high bacterial inocula (HI, is about 10^7^–10^8^ CFU/mL), i.e., significantly higher than that used in standard in vitro susceptibility (MIC) testing experiments (SI, 5 × 10^5^ CFU/mL), or whether an inoculum effect (IE) occurs. The IE describes a significant decrease in antibacterial activity of antibiotics at high bacterial inocula and is a well-known phenomenon for beta-lactams [7,8,9]. In Gram-negative bacteria, the prevalent mechanism of resistance that mediates the IE is the production of beta-lactamase enzymes. Knowledge about IE could be crucial in treating high-burden bacterial infections as lowered antibacterial activity may be responsible for unexpected antibiotic treatment failures. For example, treatment failure in patients with staphylococcal bacteremia has been reported with cefazolin due to the IE [10,11]. In another in vivo study with piperacillin/tazobactam, this combination was prone to the IE [12]. Of note, the IE has not been extensively studied with carbapenem/carbapenemase inhibitor combinations, particularly with imipenem/relebactam. In this respect, the combination of doripenem, another carbapenem antibiotic, with relebactam is also of interest.

Along with IE, another factor that can influence the in vitro activity of beta-lactam/beta-lactamase inhibitor combinations is the concentration ratio of drugs used in susceptibility testing experiments. Antibacterial activity of imipenem/relebactam is routinely determined by varying imipenem concentrations in the presence of a fixed relebactam concentration [13]. However, this traditional approach to MIC determinations for antibiotic/inhibitor combinations leads to arbitrary antibiotic-to-inhibitor concentration ratios that do not always correspond to those achieved in humans. Our group recently proposed a pharmacokinetic-based (PK-based) approach to determining MICs of antibiotic/inhibitor combinations [14]. The antibacterial effects of imipenem/relebactam and doripenem/relebactam combinations in time-kill experiments were accurately predicted by MICs determined using PK-based antibiotic-to-inhibitor concentration ratios. In addition, it was confirmed that the PK-based approach could predict the efficacy of antibiotic/antibiotic combinations in series of pharmacodynamic experiments using an in vitro dynamic model with Gram-positive [15,16,17] and Gram-negative [18] bacteria.

To explore if the IE occurs and if the carbapenem-to-inhibitor concentration ratio influences the in vitro activity of antibiotic/inhibitor combinations, we evaluated MICs of imipenem and doripenem used alone or in combination with relebactam against KPC-producing *K. pneumoniae* strains at standard and high-density inocula by the traditional method at a fixed relebactam concentration and using a pharmacokinetic-based carbapenem-to-inhibitor concentration ratio.

## 2. Materials and Methods

### 2.1. Antimicrobial Agents and Bacterial Strains

Imipenem monohydrate and doripenem hydrate powders were purchased from Acros Organics (Fair Lawn, NJ, USA). Relebactam was purchased from Invivochem (Libertyville, IL, USA). Six *bla*_KPC_-positive, by PCR, non-mucoid *K. pneumoniae* strains with different susceptibility to imipenem and doripenem were used in the study: two clinical isolates, *K. pneumoniae* 14 and 16, and four ATCC *K. pneumoniae* strains (KPC reference strains), BAA-1705, BAA-1902, BAA-1904, and BAA-1905. *K. pneumoniae* ATCC 700,603 was used as a negative control. Before each test, carbapenemase production was confirmed for each bacterial strain by a modified carbapenem-inactivation method [19].

### 2.2. Susceptibility Testing

Susceptibility testing for antibiotics and inhibitor used alone or in combination was performed using broth microdilution techniques with standard inocula of approximately 5 × 10^5^ CFU/mL (SI) and at high inocula of 5 × 10^7^ CFU/mL (HI). When used alone, MICs at SI were determined according to standard CLSI recommendations [13]. When the MICs (for single carbapenems and their combination with relebactam) were determined at HI, bacterial growth was quantified by optical density at 600 nm (OD). ODs were estimated before and after 18 h of incubation at 37 °C. The MIC was the dilution at which the 18 h OD was equal to or less than that at time 0. An inoculum effect was defined as an eight-fold or greater increase in MIC when tested with the HI relative to SI. For carbapenem/relebactam combinations, MIC testing was performed under two different conditions, as determined by method 1 or method 2 regarding the ratio of imipenem or doripenem to relebactam. Before reading, plates were incubated at 37 °C for 18 h. MIC values were obtained at least in triplicate, and the modal MICs were estimated.

Method 1 (standard, MIC_1_): MIC testing for imipenem/relebactam used a fixed relebactam concentration of 4 mg/L with doubling dilutions of imipenem according to CLSI recommendations [13]. With doripenem/relebactam, the susceptibility testing recommendations are absent, so the MIC testing procedure was the same as with imipenem/relebactam.

Method 2 (PK-based, MIC_2_): MIC testing for imipenem/relebactam and doripenem/relebactam combinations used a fixed PK-based carbapenem-to-relebactam concentration ratio of 1.5/1 by varying the carbapenem and relebactam concentrations in parallel for each subsequent dilution. This concentration ratio is equal to the therapeutic 24 h area under the concentration–time curve (AUC) ratio of imipenem or doripenem (for a 500 mg dose of each carbapenem every 6 h [20,21]) to the therapeutic AUC of relebactam (for a 250 mg dose every 6 h [21]). The PK-based ratio was equal for imipenem/relebactam and doripenem/relebactam combinations as both carbapenems are characterized by similar pharmacokinetic profiles.

The MIC breakpoints for imipenem and imipenem/relebactam susceptibility testing were used according to CLSI recommendations [22]. For the doripenem/relebactam combination, MICs were interpreted using CLSI breakpoints for doripenem. The carbapenem MIC breakpoints for HI were the same as for SI. In all cases, the interpretive criteria for susceptibility were as follows: susceptible, ≤1 mg/L; intermediate, 2 mg/L; resistant, ≥4 mg/L.

## 3. Results

### 3.1. Susceptibility Testing with Single Imipenem and Doripenem

Imipenem and doripenem MICs for carbapenemase-producing *K. pneumoniae* strains varied from 4 to 64 mg/L and from 4 to 128 mg/L, respectively, when estimated at SI (Table 1). At HI, MICs of both antibiotics were higher, and decreased susceptibility was more pronounced with imipenem (4- to 64-fold) than with doripenem (2- to 16-fold). The IE was observed in four of six *K. pneumoniae* strains exposed to imipenem and in three of six strains exposed to doripenem.

It is worth noting that 8- to 64-fold increases in carbapenem MICs at HI were observed against *K. pneumoniae* strains that were initially more susceptible to imipenem and doripenem (MICs at SI of 8 and 16 mg/L and of 4 and 8 mg/L, respectively). Less pronounced density-related MIC elevations (4-fold with imipenem and 2-fold or no increase with doripenem) occurred with two strains that were highly resistant to both carbapenems (MICs at SI of 64 or 128 mg/L).

### 3.2. Susceptibility Testing with Imipenem and Doripenem at Fixed Relebactam Concentration (Method 1)

At SI, all tested *K. pneumoniae* strains were susceptible to both carbapenems when relebactam was added, and respective MIC_1_s were 0.25 to 0.5 mg/L for imipenem and 0.06 to 1 mg/L for doripenem (Table 2).

**Figure 1:**
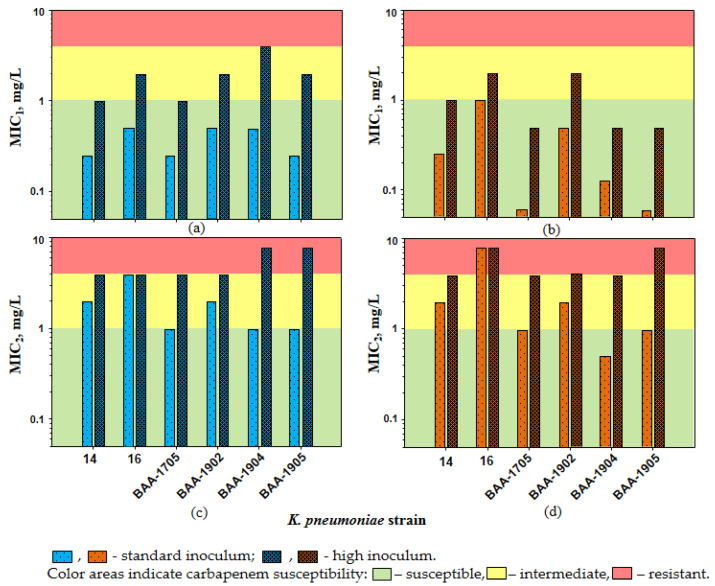
The susceptibility of *K. pneumoniae* to imipenem (**a**,**c**) and doripenem (**b**,**d**) in the presence of relebactam. The interpretive criteria for susceptibility were as follows: susceptible, ≤1 mg/L; intermediate, 2 mg/L; resistant, ≥4 mg/L.

At HI, the imipenem MICs against *K. pneumoniae* in the presence of relebactam increased 4- to 8-fold (MICs of 1–4 mg/L); as a result, for two *K. pneumoniae* strains (BAA-1904 and BAA-1905), the IE was observed. Similar results were obtained with doripenem: MICs increased 2- to 8-fold (MICs of 0.5–2 mg/L) and the IE was observed for *K. pneumoniae* strains BAA-1705 and BAA-1905. With an increase in MIC1s at HI, which was observed for all *K. pneumoniae* strains, two of six strains became imipenem-intermediate (16, BAA-1705 and BAA-1902; MIC of 2 mg/L) and one became imipenem-resistant (BAA-1904; MIC of 4 mg/L). The patterns of *K. pneumoniae* carbapenem susceptibility at HI in the presence of relebactam at fixed concentration placed alongside CLSI MIC breakpoints are shown in Figure 1a,b. With doripenem, two of six *K. pneumoniae* strains at HI had a MIC_1_ of 2 mg/L (16 and BAA-1902); therefore, intermediate carbapenem resistance was defined.

### 3.3. Susceptibility Testing at PK-Based Carbapenem-to-Relebactam Concentration Ratio (Method 2)

At SI, MIC_2_s were higher than MIC_1_s for all *K. pneumoniae* strains: 1–4 versus 0.25–0.5 mg/L with imipenem and 0.5–8 versus 0.06–1 mg/L with doripenem (Table 2). As seen in the Table, when exposed to imipenem or doripenem combined with relebactam, three of six *K. pneumoniae* strains had MIC_2_ values that were classified as carbapenem-intermediate (MIC of 2 mg/L for 14 and BAA-1902) or resistant (MIC of 4 and 8 mg/L for 16).

At HI, density-related MIC_2_ elevations led to even lower *K. pneumoniae* carbapenem susceptibility, and all strains became imipenem- or doripenem-intermediate or resistant (Figure 1c,d and Table 2). Similar to MIC_1_s, MIC_2_s at SI and HI reached an 8-fold difference and the IE was detected for two *K. pneumoniae* strains (BAA-1904 and BAA-1905) with both carbapenems.

## 4. Discussion

In the current study, carbapenemase-producing *K. pneumoniae* strains were exposed to imipenem and doripenem at HI (5 × 10^7^ CFU/mL) and the IE was observed against strains with initial carbapenem MICs of 4 to 16 mg/L. MIC elevations were 8- to 64-fold. Carbapenem susceptibility of *K. pneumoniae* strains highly resistant to imipenem and doripenem (MICs 64 and 128 mg/L at SI) also decreased at HI, but the IE was not observed. It is possible that the carbapenemase enzymes may be potentiated more at increased inocula in strains with lower MICs than in highly resistant strains.

**Figure 2:**
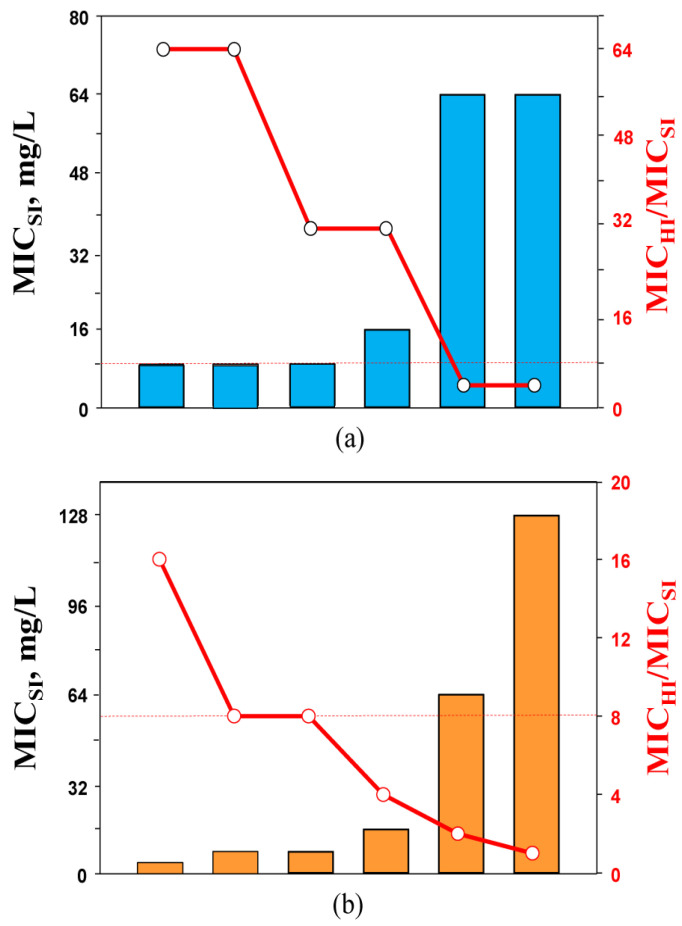
The MICs of imipenem (**a**) and doripenem (**b**) against *K. pneumoniae* at standard inoculum density (bars) and respective changes in the susceptibility at high inoculum density (circles). The dashed lines indicate the lower level of MIC changes associated with the inoculum effect (IE, ≥8-fold).

Assuming these patterns of carbapenem susceptibility at HI, we examined whether there is any relationship between the bacterial susceptibility at SI and the exhibited elevations in MIC at high inocula. Imipenem and doripenem MICs against *K. pneumoniae* strains at SI were compared with the respective inoculum-related MIC changes and were expressed as the ratio of antibiotic MIC at HI to MIC at SI—MIC_HI_/MIC_SI_. As seen in Figure 2, there was a clear relationship: the lower the imipenem and doripenem MICs at SI, the greater the respective MIC elevations at HI. In general, imipenem alone was more prone to the IE than doripenem, as imipenem MIC elevations associated with the IE were always higher. Similar imipenem inoculum-related MIC increases (32-fold and higher) for KPC-producing *K. pneumoniae* strains were described in several in vitro studies [23,24].

Carbapenem MICs decreased significantly in the presence of relebactam both at SI and HI. At the same time, bacterial susceptibility to antibiotic/inhibitor combinations differed between these inocula. At HI, the 2- to 8-fold MIC elevations were detected compared to respective combination MICs at SI. However, IE was observed for only two *K. pneumoniae* strains regardless of the combination MIC testing method. In previous studies, diminished activity at high inocula was reported for piperacillin/tazobactam [25,26,27,28], amoxicillin/clavulanate [26], ampicillin/sulbactam and ticarcillin/clavulanate [25], ceftazidime/avibactam [29,30], aztreonam/avibactam [30], and several cephalosporin/clavulanate combinations [31] against *Enterobacteriaceae*, including *K. pneumoniae*. Piperacillin/tazobactam, ticarcillin/clavulanate, and aztreonam/avibactam combinations were prone to the inoculum effect, while for with other antibiotic/inhibitor combinations, the IE was not registered at all (ceftazidime/avibactam) or was observed only for a minority of tested isolates. Our data with imipenem/relebactam and doripenem/relebactam combinations demonstrated similar patterns.

Although in the current study the IE was observed only in some tested *K. pneumoniae* strains, density-related combination MIC elevations (2- to 8-fold) were found in most of them. Thus, at high inocula, carbapenem MICs in the presence of relebactam (especially when estimated at the pharmacokinetic-based concentration ratio) increased to levels associated with carbapenem resistance (according to the CLSI breakpoints [22]). Hence, even slightly diminished activity of carbapenem/relebactam combinations at high inocula (i.e., less than 8-fold) should be considered a risk factor for decreased treatment efficacy of high-burden bacterial infections and possible treatment failure.

**Figure 3:**
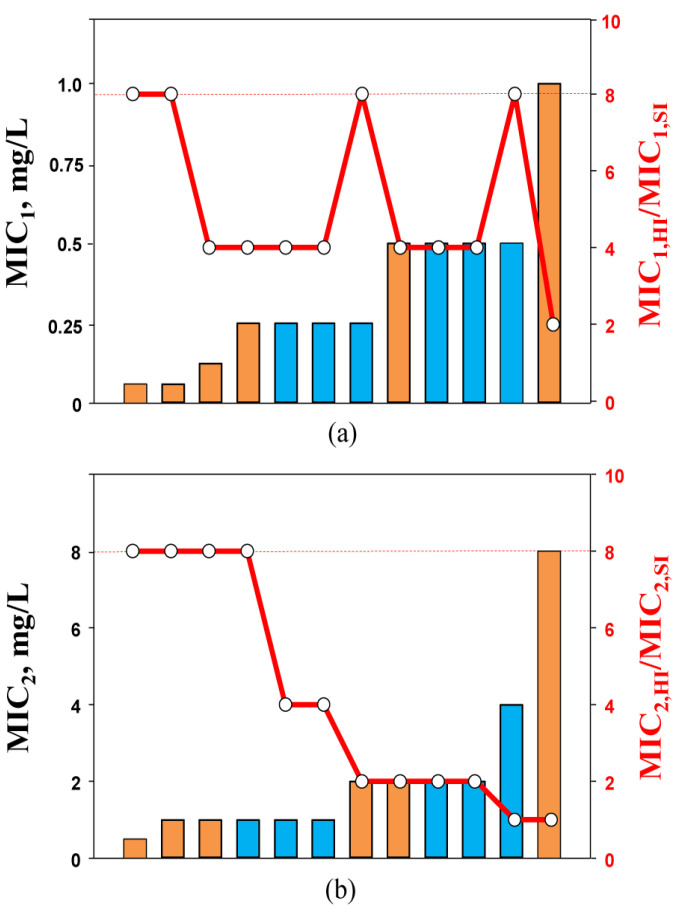
MIC_1_ (**a**) and MIC_2_ (**b**) values of imipenem (blue bars) and doripenem (orange bars) in the presence of relebactam against *K. pneumoniae* at standard inoculum density and changes in the susceptibility at high inoculum density (circles). The dashed lines indicate the lower level of MIC changes associated with the inoculum effect (IE, ≥8-fold).

It would be interesting to know if the decrease in carbapenem susceptibility in the presence of relebactam at increased inocula related to MICs at SI, similar to that observed with single imipenem and doripenem, could be found. To answer this question, the combination MICs at SI against *K. pneumoniae* strains determined by two methods were compared with the respective inoculum-related MIC changes (expressed as ratio of MIC_1_ or MIC_2_ at HI to MIC_1_ or MIC_2_ at SI—MIC_1_,_HI_/MIC_1_,_SI_ or MIC_2_,_HI_/MIC_2_,_SI_, respectively). As seen in Figure 3a, consistency between the carbapenem/inhibitor MIC_1_s at SI and the MIC_1_,_HI_/MIC_1_,_SI_ ratio was not observed. Similar observations were made in other in vitro studies with Gram-negative bacteria, and several combinations of cephalosporin/clavulanate [31] and piperacillin/tazobactam [26] (all had a fixed inhibitor concentration) were analyzed. There was no relationship between the density-related MIC elevations and the susceptibility of *K. pneumoniae* or *Escherichia coli* to tested beta-lactam/beta-lactamase inhibitor combinations at SI.

However, a relation was found between MIC_2_s (at SI) and the respective MIC_2_,_HI_/MIC_2_,_SI_ ratio (Figure 3b): the higher the carbapenem/relebactam MIC_2_ at SI, the less likely that density-related MIC_2_ changes would be observed. With *K. pneumoniae* strains for which MIC_2_s at SI were highest (4 or 8 mg/L), no diminished activity of the combination was detected at HI. Therefore, with imipenem/relebactam and doripenem/relebactam combinations, similar susceptibility patterns at HI were observed as with imipenem and doripenem alone. This could likely be explained by the inoculum-potentiated beta-lactamases in low-MIC strains that are not blocked by the same inhibitor concentration at SI. It is possible that alternative resistance mechanisms could contribute to resistance to imipenem and doripenem combinations with relebactam at HI. For example, in another in vitro study with KPC-producing *K. pneumoniae* that exhibited a carbapenem-susceptibility phenotype, rapid inoculum-induced high-level imipenem resistance was mediated by the essential coordination between *bla*_KPC_ and *OmpK*36 expression [23]. Similar resistance mechanisms with coordination of *bla*_KPC_ and mutations affecting the genes encoding porins were found in KPC-producing *K. pneumoniae* isolates with decreased susceptibility to imipenem/relebactam combinations (MIC of 2 mg/L or higher). Moreover, the same mechanisms of *K. pneumoniae* resistance to combinations of imipenem with relebactam were also described in vivo. It was reported that the *K. pneumoniae* clinical isolate initially susceptible to imipenem/relebactam acquired resistance to this combination during antimicrobial therapy of a hematology patient with a bloodstream infection [32]. The resistance mechanism of this *K. pneumoniae* isolate was associated with an increased *bla*_KPC-3_ copy number and disruptions of porins (*OmpK*35 and *OmpK*36).

The relation between the MIC_2_ at SI and the MIC_2_,_HI_/MIC_2_,_SI_ ratio indicates that the use of a PK-based approach to carbapenem/inhibitor combination MIC determination predicts MIC elevations at HI and the probability of the IE in carbapenemase-producing *K. pneumoniae*.

In our previous study [14], the predictive potential of MIC testing at PK-based carbapenem-to-relebactam concentration ratios regarding the antibacterial effectiveness of imipenem/relebactam and doripenem/relebactam combinations against KPC-producing *K. pneumoniae* was demonstrated. In contrast to the standard approach to MIC determination using a fixed relebactam concentration, the PK-based approach allowed a significant correlation between the C/MIC parameter (the ratio of carbapenem concentration in the time-kill experiments and the culture MIC) and the antibacterial effect. These data suggest that MIC determinations at PK-based carbapenem-to-relebactam concentration ratios might be better in vitro predictors of antibacterial effects than MICs determined at a fixed concentration of relebactam, i.e., at an arbitrary antibiotic/inhibitor ratio. We believe that the PK-based approach could be a reliable tool for susceptibility testing of carbapenemase-producing *K. pneumoniae* strains at both SI and HI. Pharmacodynamic experiments with imipenem/relebactam and doripenem/relebactam combinations at SI and HI in in vitro dynamic models are needed to further validate the PK-based approach to determine carbapenem/relebactam MIC and IE determinations with different bacterial inocula.

Our study has several limitations. It did not include many *K. pneumoniae* strains, and additional studies with other *K. pneumoniae* isolates and with other Gram-negative bacteria are necessary to fully evaluate the IE using standard methods and the PK-based approach. Moreover, we did not determine if the *K. pneumoniae* strains used in the study are biofilm producers. Previously, it was reported that this bacterial feature could significantly influence the antibiotic susceptibility and treatment outcome [33]. This limits the potential clinical relevance of our findings. In addition, the subsequent studies with a wide range of antibiotic/inhibitor combinations would enhance the generalizability of our results. We did not investigate the resistance mechanisms exhibited by *K. pneumoniae* strains at increased inocula to confirm our assumptions about prevalent mechanisms of imipenem/relebactam and doripenem/relebactam resistance at HI.

## 5. Conclusions

In the current study, we evaluated the IE with carbapenem/carbapenemase inhibitor combinations. Previously, the IE was shown to appear with beta-lactams and beta-lactam/beta-lactamase combinations. According to our findings, the IE was observed with both carbapenem/relebactam combinations regardless of the MIC testing method; however, IE was not prevalent among the tested KPC-producing *K. pneumoniae* strains. It seems promising that relebactam can decrease inoculum-related susceptibility reductions and minimize the impact of the IE. At the same time, although imipenem/relebactam and doripenem/relebactam combinations were less prone to the IE compared to single antibiotics, at the HI, carbapenem MICs in the presence of relebactam increased to levels associated with carbapenem resistance (especially with the PK-based concentration ratio). In the current study, we also reported that the use of a PK-based approach to carbapenem/inhibitor combination MIC determinations allows the prediction of MIC elevations at HI and the probability of the IE in carbapenemase-producing *K. pneumoniae* strains. Accordingly, it can be hypothesized that using the PK-based approach could allow a more realistic assessment of carbapenem susceptibility in KPC-producing *K. pneumoniae* strains and might be a helpful option to evaluate treatment failures due to IE with carbapenem/carbapenemase inhibitor combinations.

## Figures and Tables

**Table 1:** MICs (mg/L) of imipenem and doripenem at standard inoculum (SI) and high inoculum (HI) density against *K. pneumoniae*.

*K. pneumoniae* Strain	Imipenem			Doripenem		
	MIC_SI_	MIC_HI_	MIC_HI_/MIC_SI_	MIC_SI_	MIC_HI_	MIC_HI_/MIC_SI_
14	16	512	32 *	16	64	4
16	64	256	4	128	128	1
BAA-1705	8	256	32 *	8	64	8 *
BAA-1902	64	256	4	64	128	2
BAA-1904	8	512	64 *	4	64	16 *
BAA-1905	8	512	64 *	8	64	8 *

* MIC changes associated with the inoculum effect (IE).

**Table 2:** MICs (mg/L) of imipenem and doripenem in combination with relebactam at standard inoculum (SI) and high inoculum (HI) density against *K. pneumoniae*.

*K. pneumoniae* Strain	Imipenem/Relebactam			Doripenem/Relebactam		
	MIC_1,SI_	MIC_1,HI_	MIC_1,HI_/MIC_1,SI_	MIC_1,SI_	MIC_1,HI_	MIC_1,HI_/MIC_1,SI_
14	0.25	1	4	0.25	1	4
16	0.5	2	4	1	2	2
BAA-1705	0.25	1	4	0.06	0.5	8 *
BAA-1902	0.5	2	4	0.5	2	4
BAA-1904	0.5	4	8 *	0.125	0.5	4
BAA-1905	0.25	2	8 *	0.06	0.5	8 *
	**MIC_2,SI_**	**MIC_2,HI_**	**MIC_2,HI_/MIC_2,SI_**	**MIC_2,SI_**	**MIC_2,HI_**	**MIC_2,HI_/MIC_2,SI_**
14	2	4	2	2	4	2
16	4	4	1	8	8	1
BAA-1705	1	4	4	1	4	4
BAA-1902	2	4	2	2	4	2
BAA-1904	1	8	8 *	0.5	4	8 *
BAA-1905	1	8	8 *	1	8	8 *

* MIC changes associated with the inoculum effect (IE).

## Data Availability

Not applicable.
